# Genome evolution drives transcriptomic and phenotypic adaptation in *Pseudomonas aeruginosa* during 20 years of infection

**DOI:** 10.1099/mgen.0.000681

**Published:** 2021-11-26

**Authors:** Samuel J.T. Wardell, Jeff Gauthier, Lois W. Martin, Marianne Potvin, Ben Brockway, Roger C. Levesque, Iain L. Lamont

**Affiliations:** ^1^​ Department of Biochemistry, University of Otago, Dunedin, New Zealand; ^2^​ Institut de biologie intégrative et des Systèmes, Université Laval, Québec, Canada; ^3^​ Department of Medicine, University of Otago, Dunedin, New Zealand

**Keywords:** adaptive gene expression, antibiotic resistance, genetic bottleneck, genome evolution, genome deletion, mutator strain

## Abstract

The opportunistic pathogen *Pseudomonas aeruginosa* chronically infects the lungs of patients with cystic fibrosis (CF). During infection the bacteria evolve and adapt to the lung environment. Here we use genomic, transcriptomic and phenotypic approaches to compare multiple isolates of *P. aeruginosa* collected more than 20 years apart during a chronic infection in a CF patient. Complete genome sequencing of the isolates, using short- and long-read technologies, showed that a genetic bottleneck occurred during infection and was followed by diversification of the bacteria. A 125 kb deletion, an 0.9 Mb inversion and hundreds of smaller mutations occurred during evolution of the bacteria in the lung, with an average rate of 17 mutations per year. Many of the mutated genes are associated with infection or antibiotic resistance. RNA sequencing was used to compare the transcriptomes of an earlier and a later isolate. Substantial reprogramming of the transcriptional network had occurred, affecting multiple genes that contribute to continuing infection. Changes included greatly reduced expression of flagellar machinery and increased expression of genes for nutrient acquisition and biofilm formation, as well as altered expression of a large number of genes of unknown function. Phenotypic studies showed that most later isolates had increased cell adherence and antibiotic resistance, reduced motility, and reduced production of pyoverdine (an iron-scavenging siderophore), consistent with genomic and transcriptomic data. The approach of integrating genomic, transcriptomic and phenotypic analyses reveals, and helps to explain, the plethora of changes that *P. aeruginosa* undergoes to enable it to adapt to the environment of the CF lung during a chronic infection.

## Impact Statement

*Pseudomonas aeruginosa* is a bacterium that causes serious and often fatal infections in already ill people, such as those with the genetic disease cystic fibrosis (CF). *P. aeruginosa* infects the airways of people with CF, impairing lung function, reducing quality of life and shortening life expectancy. Infections can last for years or even decades, during which time the bacteria adapt and evolve to maximize their survival and growth in the lungs. To better understand this process we studied samples of *P. aeruginosa* from a CF patient who was infected with these bacteria for more than 20 years. We found that during the infection the genomes of the bacteria underwent many changes in genes affecting multiple properties, such as antibiotic resistance, metabolism, mobility and adherence. We compared expression of all of the genes in isolates obtained 20 years apart and found that the genome changes caused altered expression of very many genes. Lastly, we showed that genome and gene expression changes resulted in biological changes that helped the bacteria survive during the infection. This research advances understanding of how *P. aeruginosa* changes to successfully colonize the lung during infection, and may help develop new treatment strategies.

## Data Summary

All raw fastq files from the 15 whole genome sequences and six RNA sequencing samples are available under BioProject accession number PRJNA588274. Genome accession numbers are listed in Table S3. The authors confirm that all supporting data and protocols have been provided within the article and through the supplementary data files.

## Introduction

*Pseudomonas aeruginosa* is a Gram-negative bacterium present in a range of environments. It is also an opportunistic human pathogen, commonly infecting immune-compromised patients [1, 2]. *P. aeruginosa* is a major cause of increased morbidity in individuals with cystic fibrosis (CF), where it colonizes the lungs [2]. Patients are typically infected by *P. aeruginosa* from the environment, which can act as a reservoir for infection [3]. Infections in CF patients commonly become chronic, with the infecting bacteria adapting to survive antibiotic treatment and the environment of the host lung [4, 5].

In *P. aeruginosa *, antibiotic resistance is multifactorial with a range of genetic events responsible for the development of resistance [6, 7]. Mutations altering target proteins reduce the binding affinities of antibiotics. For example, mutations in *gyrA* or *gyrB* that encode subunits of DNA gyrase lead to fluoroquinolone resistance [8], and mutations in *ftsI* that encodes penicillin-binding protein 3 contribute to meropenem resistance [9, 10]. Decreased uptake of antibiotics can occur due to changes in membrane permeability. For example, mutations in *oprD* that encodes a porin contribute to carbapenem resistance [11] and changes to lipopolysaccharide (LPS) increase tolerance to polymyxins and aminoglycosides [12, 13]. Increased antibiotic efflux can arise due to increased expression of efflux systems, arising from mutations affecting regulatory proteins such as MexZ [6], and increased production of AmpC β-lactamase can result from mutations in genes such as *mpl* [6, 14]. Although antibiotic resistance in *P. aeruginosa* in CF predominantly occurs through mutations in chromosomally encoded genes, horizontal gene transfer can also introduce genes that confer a resistant phenotype [15, 16].

As well as resisting antibiotic treatment, during chronic infection in CF patients *P. aeruginosa* adapts to other challenges of the lung environment, including oxidative stress, the immune system, host withholding of micronutrients such as iron and zinc, and competition from other microbiota [17, 18]. The bacteria can undergo multiple phenotypic changes including changes to metabolism, loss of motility, increased production of the extracellular polysaccharide alginate, reduced virulence factor and siderophore production, emergence of auxotrophs, and occurrence of small colony variants and hypermutator strains [4, 5, 19–25]. The advent of high-throughput DNA sequencing technologies has allowed analysis of the genetic changes that underlie phenotypic changes during *P. aeruginosa* infections in CF. The first such study identified multiple mutations associated with antibiotic resistance and immune evasion over the course of 8 years of infection [26]. Subsequent studies have also followed the progression of genomic changes undergone by *P. aeruginosa* during chronic infection in CF [19, 21, 22, 27–31] and non-CF patients [32], including gene loss and acquisition [33], and have identified many other genes that undergo mutation during the course of infection. Some studies have also investigated associated transcriptomic and phenotypic changes [22, 34, 35]. However, our understanding of the relationship between genome-wide genetic changes, consequent changes to the transcriptome and the resulting phenotypic changes that facilitate adaptation to the lung environment is limited. The aim of this study, therefore, was to investigate in detail the evolution of *P. aeruginosa* during infection over the course of 20 years in the lungs of a patient with CF, comparing multiple isolates and merging phenotypic, transcriptional and genotypic changes to understand how *P. aeruginosa* evolved during the course of the infection.

## Methods

### Sample isolation

Multiple sputum samples were collected from a single patient located in Dunedin, New Zealand, in 1991 when the patient was aged 26 years, and from the same patient in 2011–2012. The patient was male with CF genotype Δ508/Δ508. Single *P. aeruginosa* colonies were obtained from sputum inoculated onto cetrimide agar [36]. A summary of lung function is provided in Table S1 (available in the online version of this article) and a summary of antibiotics given to the patient between first and last sampling is given in Table S2.

### Minimum inhibitory concentration

Antibiotic resistance of all strains to multiple antibiotics were determined on Difco Mueller-Hinton (MH) agar [37]. Colonies were selected and grown in Difco MH broth. Overnight cultures were serially diluted and plated using an inoculum of approximately 10^4^ c.f.u. per spot onto MH agar containing doubling concentrations of antibiotics. The minimum inhibitory concentration (MIC) was recorded as the lowest concentration of antibiotic that inhibited bacterial growth (mg l^−1^). Susceptibilities for meropenem (Penembact; Venus Remedies), tobramycin (Mylan New Zealand) and ciprofloxacin (Cipflox; Mylan New Zealand) were tested in duplicate.

### Swimming motility

Motility of the bacteria was assessed using a previously described method [38]. In short, M8 medium containing 0.3 % agar was inoculated by stabbing into the agar with a pipette tip that had been dipped in culture (with an OD_600nm_ of 1.0) and incubated at 37 °C for 24 h. Plates were then imaged and the area of motility was determined using ImageJ [39].

### Quantification of pyoverdine production

Bacteria were grown in King’s B broth [40] in 96-well clear-bottomed, black walled tissue culture plates (Corning). The microtitre plates were incubated in a BMG FLUOstar Omega microplate reader at 37 °C with shaking at 200 r.p.m. for 24 h. Optical density (OD_600nm_) was recorded every 30 min, to monitor the growth of the isolates. Simultaneously, pyoverdine was measured spectrophotometrically using a fluorescence assay, with excitation at 410 nm and emission at 460 nm. The pyoverdine production values used were the fluorescence after 24 h normalized to the OD_600_ of the final culture.

### Biofilm formation assay

Measurement of biofilm formation via crystal violet staining was performed as described previously [41, 42]. In short, isolates were grown in M63 medium supplemented with 0.4 % arginine to promote biofilm formation [42]. Cultures were grown for 24 h in U-bottomed 96-well plates (Corning), at 37 °C without shaking, and OD_600nm_ was measured. Wells were washed out with water to remove planktonic cells and biofilms were stained with crystal violet. Acetic acid was used to dissolve the stain into an aqueous solution for measurement. Measurements were made at 550 nm and normalized to the OD_600nm_ prior to staining.

### Growth rate assay

Growth of bacterial cultures in LB or synthetic CF nedium (SCFM) [43] in 96-well plates, incubated at 37 °C with shaking, was measured as described previously [9]. The OD_600_ was measured every 30 min for 18 h for growth in LB, and for 24 h in SCFM.

### DNA extraction and quality check

An isolated colony was inoculated in brain heart infusion (BHI) broth and grown at 35 °C without agitation. Cells were harvested by centrifugation for 10 min at 5000 ***g*** to have approximately 2×10^9^ cells per pellet. Supernatant was discarded and pellets were placed at −80 °C until DNA extraction. DNA extractions were performed using DNeasy Blood and Tissue kit (Qiagen) following the manufacturer’s protocol, with the following additional steps. First, to avoid DNA shearing, all vortexing steps were replaced by tube inversions. After the bacterial lysis step, RNA was digested by adding 10 µl of RNase cocktail (Invitrogen) to the lysate and incubating at room temperature for 5 min. A second washing with 500 µl of AW2 buffer was performed instead of one wash. Finally, DNA was eluted twice with 50 µl of 10 mM Tris-HCl pH 8. Extracted DNA was quantified by the Qubit dsDNA BR method (Invitrogen) and purity (based on 260/280 and 260/230 absorbance ratios) was measured with a Nanodrop 2000 spectrophotometer (Thermo Fisher).

### Illumina MiSeq genome analysis

Short paired-end reads (2×300 bp) were obtained for each sequenced strain by Illumina MiSeq sequencing as previously described [44, 45]. Paired-end sequencing reads were checked using FastQC version 0.11.5. Trimmomatic version 0.36 [46] was used to filter the raw Illumina short reads and remove adapter sequences. Post-trimming, all reads used subsequently were at least 20 nt long, with a minimum average phred score of >20, and paired. Draft genome assemblies were created using SPAdes version 3.12.0 [47] (Table S3).

### Oxford Nanopore sequencing

One microgram of high-molecular-weight genomic DNA from each *P. aeruginosa* isolate was transferred into a 96-well microtitre PCR plate and volumes were adjusted to 48 µl using nuclease-free water. DNA repair and end-prep and native barcode ligation were performed as in Oxford Nanopore’s instructions for native barcoding genomic DNA version NBE_9065_v 109_revG_23May2018 using an LSK-109 sequencing kit, native barcode expansions and reagents (New England Biolabs) specified in the protocol. Barcoded and purified DNA was quantified with Qubit and 50 ng of each were pooled together to obtain ca. 900 ng of DNA in the pool. Volume was adjusted to 65 µl with nuclease-free water. Adapter ligation and clean-up were performed following the manufacturer’s instructions. Pooled libraries were purified with SparQ PureMag beads (QuantaBio) and eluted by suspending beads in 14 µl of 10 mM Tris-HCl (pH 8) on the MixMate (24 °C, 10 min, 1000 r.p.m.). One microlitre of eluate was kept for Qubit quantification. A total of 379 ng of pooled libraries was loaded on a MinION FLO-MIN106 flow cell (v. R9.4). Raw signal acquisition was performed with Oxford Nanopore’s proprietary software MinKNOW version 8.3.1 (Oxford Nanopore Technologies), with the basecalling option turned off, run length set at 72 h and time between mux scans set at 3 h. The raw sequencing data (in fast5 format) were basecalled and demultiplexed post-sequencing using Guppy v.4.2.2-gpu [48].

### Hybrid genome assembly

Long nanopore reads of over 1000 bp in length and mean Phred score >9 were selected with NanoFilt v2.3.0 [49] and adapter sequences were trimmed with poreChop v0.2.4 (https://github.com/rrwick/Porechop). The Unicycler assembly pipeline v0.4.8 [50] was used to perform a hybrid genome assembly in which Illumina short reads were assembled into contigs and then bridged with MinION long reads to form complete and circular replicons. Assembly statistics (number of contigs, total bases, longest contigs, length N50, etc.) were calculated and compiled using assembly-stats v1.0.1 (https://github.com/sanger-pathogens/assembly-stats) and in-house Unix shell scripts.

### Core genome analysis

To generate a core genome (defined as DNA that is present in all isolates), complete genome assemblies were compared using parsnp version 1.2 from the Harvest suite 1.1.2 [51]. E-S2239-15 was excluded from core genome generation as it is a genetic outlier. The core genome covered on average 96 % of the remaining assemblies. Genome comparisons used for the generation of Figs 2 and S1 were visualized using gingr version 1.2 from the Harvest suite, and an SNP cladogram from the differences between isolates was visualized using FigTree version 1.4.4 [52]. Assemblies were compared using BRIG version 0.95 [53]. Gene prediction was performed on genome assemblies using Prokka version 1.13 [54] using bacterial annotation and PAO1 homologue gene IDs as PAO1 is the most closely related reference strain. Multi-locus sequence typing (MLST) for each isolate was determined using pubMLST [55].

### Novel region discovery

To identify new regions of DNA obtained from the transition of earlier to later isolates, Panseq version 3.1.0 [56] was used. Novel regions with a minimum length of 500 bp were identified by comparing early isolate E-S2239-16 with all other isolates. To identify any resistance-associated genes that may have been acquired by horizontal gene transfer, ResFinder version 2.3 [57] was used.

### Variant analysis

Indels, duplications and small variants were analysed as previously described [9], using Breseq version 0.30.0 [58] with E-S2239-16 as a reference for all analyses. All Breseq outputs (variants called between the isolate of interest and E-S2239-16) were extracted and compared to each other using GDtools-GDcompare as part of the Breseq package.

### RNA sequencing (RNAseq) analysis

Three biological replicate cultures of representative isolates E-S2239-16 and L-001–1C were grown in SCFM to exponential growth phase (OD_600nm_ of 0.4–0.6) and RNA was extracted using the GeneJET RNA Purification kit, according to the manufacturer’s protocol. RNAseq ScriptSeq libraries were prepared from rRNA-depleted RNA samples using a ScriptSeq Complete Bacterial Kit and sequenced as 125 bp paired-end reads (average of 15 million reads per sample) using an Illumina HiSeq 2000 by New Zealand Genomics.

Reads were trimmed using Trimmomatic as for whole-genome sequencing. Trimmed reads were mapped onto the annotated E-S2239-16 genome using Kalisto version 0.44.0 [59], using 1000 bootstraps (Table S4). Differential gene expression was assessed using Sleuth version 0.30.0 [60] in R version 3.6.0 [61] (Fig. S2). A log_2_ transformation function was done in Sleuth to output log_2_ fold change between E-S2239-16 and L-001–1C, and a false discovery rate (FDR)-adjusted *P* value of ≤0.01 was considered to indicate significantly differentially expressed.

## Results

### A single *P. aeruginosa* lineage extends through a 20-year infection

**Fig. 1.**
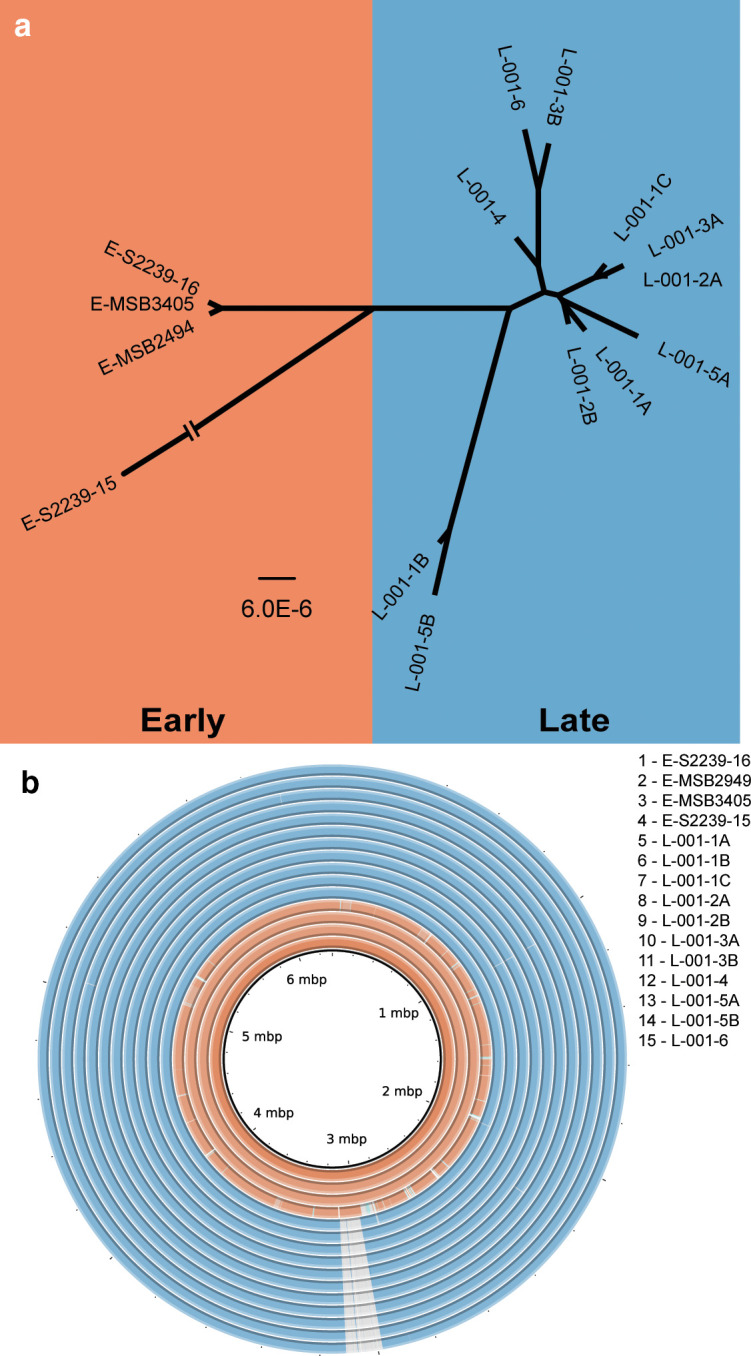
Whole genome analysis of earlier and later isolates. (**a**) Dendogram representing relationships between isolates, with branch lengths representing the number of nucleotide differences (bar, 6.0×10^−6^ nt differences per site). The strain E-S2239-15 branch is not to scale as it is distinct from all other isolates (Fig. 1). (**b**) Genome comparison of earlier (orange) and later (blue) isolates. Genomes were aligned using BRIG with the innermost ring being reference strain E-S2239-16. Grey hatching indicates absence of DNA that is present in the reference strain. Regions in darker shades are 100 % identical, with a lighter shade being 99 % identical.

Four earlier (1991 – sample prefix E) and 11 later (2012–2013 – sample prefix L) isolates of *P. aeruginosa* were obtained from an individual with CF (Table 1, Table S1). The patient suffered a decline in lung function, as shown by reduced forced expiratory volume (FEV1) and indicating advancing lung pathology, between collection dates of the earlier and later samples (Table S1). The patient was treated with a wide range of antibiotics (Tables S1 and S2) and the later isolates had on average higher antibiotic resistance than the earlier isolates (Table 1). The complete genome sequences of the isolates were determined, and details of genome assemblies are given in Table S3. A cladogram was reconstructed based on the DNA shared between isolates (Fig. 1a; Fig. S1). This showed that one isolate, E-S2239-15, is a genetic outlier (MLST type 110) and the remaining isolates are clonal (MLST type 244). The three other earlier isolates are closely related, with a median of 12 non-synonymous differences and 12 synonymous or intergenic differences (Tables S5 and S6). The later isolates are genetically very similar to three of the earlier isolates (Fig. 1b; Fig. S1; Tables S5 and S6) although they had higher genetic diversity (median of 190 non-synonymous differences for all pairwise comparisons between the later isolates) than the three clonal earlier isolates. There was no evidence of later isolates descended from E-S2239-15, nor was there any evidence of isolates that had been independently acquired after the earlier infecting strains. These findings indicate the same infecting lineage was present in the patient for over 20 years.

**Table 1.** Antibiotic minimum inhibitory concentrations (mg l^–1^) of isolates in this study

Isolate	Collection date (MM/YY)	Ciprofloxacin	Meropenem	Tobramycin	Ceftazidime
E-S2239-16	12/91	0.5	0.25	0.25	1
E-MSB2494	12/91	1*	0.25	0.25	1
E-MSB3405	12/91	**1**	0.125	0.25	0.5
E-S2239-15	12/91	**1**	1	2	1
L-001–1A	08/12	**2**	**16**	**8**	4
L-001-1B	08/12	**4**	**16**	2	**16**
L-001–1C	08/12	**2**	8	**8**	4
L-001–2A	10/12	**2**	8	**8**	**8**
L-001-2B	10/12	**2**	8	**8**	4
L-001–3A	11/12	**2**	8	4	4
L-001-3B	11/12	**2**	8	2	4
L-001–4	01/13	**2**	8	2	4
L-001–5A	04/13	**1**	**16**	4	4
L-001-5B	04/13	**2**	8	2	**16**
L-001–6	05/13	**2**	**16**	2	**8**

*Antibiotic resistance is shown in bold type. Resistance breakpoints (eucast.org) are: ciprofloxacin, >0.5; meropenem, >8; tobramycin, >2, ceftazidime, >8.

Acquisition and loss of DNA in the later isolates was analysed. All later isolates have lost a 125 kb region of the genome (Fig. 1b). This region includes 130 predicted genes, corresponding to PA2229–PA2359 in *P. aeruginosa* PAO1. A list of the deleted genes is included in Table S7. The deleted region overlaps with deletions that have occurred on multiple occasions during chronic *P. aeruginosa* infections in patients with CF or non-CF bronchiectasis [32, 62]. Many of the deleted genes encode regulatory proteins or enzymes that are involved in catabolic processes and nutrient acquisition, reflecting the myriad metabolic changes that occur in *P. aeruginosa* during adaptation to the nutrient conditions of the CF lung environment [63]. Genes required for synthesis of *psl* polysaccharide, one of three extracellular polysaccharides that can be made by *P. aeruginosa* and that influences biofilm formation *in vitro* [64–66], are also deleted. The loss of *psl* genes, in multiple isolates, suggests significant differences between biofilm architecture *in vitro*, and during long-term infection in the lung. The *amb* genes required for synthesis of a toxin, aminomethoxybutenoic acid (AMB), by *P. aeruginosa* [67] have also been lost, consistent with reduced virulence that is often associated with isolates from long-term infections [23].

All later isolates have a sequence of approximately 44 kb that shares 98 % identity to *Pseudomonas* phage LKA5. The absence of this phage from earlier isolates indicates that it was acquired during infection. We did not identify any acquired genes associated with antibiotic resistance or with known roles in pathogenicity.

Four later isolates also had an inversion of a large (0.9 Mb) portion of the genome relative to the earlier isolates (Fig. S3). Recombination endpoints were in rRNA (rrn) operons, which provide repeat sequences that can serve as inversion endpoints in *P. aeruginosa* [68]. Genomic inversions have been reported previously in isolates of *P. aeruginosa* from long-term infections in individuals with CF [69], although their impact of the bacteria is not fully understood.

### Genetic differences between isolates reveal mutations that enhance infectivity

Identification of all genetic differences between the 15 clonal isolates was performed using earlier isolate E-S2239-16 as a reference. A total of 892 different variants were identified across all 15 isolates with an average of 298 variants per isolate, and an average of 321 mutations per late isolate (Table S5). Of these 892 variants, 562 were non-synonymous differences that affected a total of 515 genes. The remaining variants were either synonymous or changes in intergenic regions of the genome. In total, 552 non-synonymous variants were only present in later isolates and are very likely to be mutations that arose during infection. The later isolates showed a mutation rate of approximately 16 mutations per year relative to the earlier isolates, although this value may be an underestimate due to the probable population bottleneck between the earlier and later isolates.

Later isolates in our study contain a different variant than ancestral isolates (M185K) in the *mutY* gene. The MutY protein reduces the frequency of GC→TA transversions that can arise following oxidation of guanine bases in DNA, and *mutY* mutants of *P. aeruginosa* have up to a 7.5-fold higher mutation rate than the wild-type [70]. A high proportion of the single nucleotide mutations (79.2%) were GC→TA transversions (Table S5; Table S8) so that it is likely that the MutY M185K variant has reduced or no function.

A large number of genes can undergo mutation to increase antibiotic tolerance as *P. aeruginosa* evolves during infection in CF [19, 22, 28, 71]. Comparison of earlier and later isolates showed that many of these genes underwent mutation during infection (Table 2; Table S5). For example, resistance to meropenem is associated with mutations in the porin-encoding gene *oprD* [72], and all later isolates had a nonsense mutation in this gene. Most of the later isolates also had at least one mutation in the *ftsI* gene that encodes a meropenem target protein, PBP3 [73, 74], and mutations in this gene also contribute to meropenem resistance [10]. These findings are consistent with the higher MIC values of all later isolates for meropenem (Table 1). All later isolates also have a mutation in *fusA1* that encodes elongation factor G, and nine later isolates have a mutation in *fusA2*. Consistent with the higher MICs of later isolates for tobramycin, mutations in *fusA1* confer resistance to aminoglycosides [28, 75] and *fusA2* mutations have also been suggested to be involved in aminoglycoside resistance [76]. Later isolates also have higher MICs for ciprofloxacin, and these isolates contained a DNA gyrase mutation (*gyrB* E469D) and mutations in *gyrB* are associated with fluoroquinolone resistance [8]. Curiously, none of the later isolates had mutations in *gyrA* associated with fluoroquinolone resistance, whereas two of the earlier isolates contained a *gyrA* variant (T83A) that increases tolerance to fluoroquinolones [8, 10, 77–79]. Lastly, mutations in *mpl* contribute to ceftazidime resistance [80] and all later isolates have a mutation in this gene and higher MICs for ceftazidime. Overall, the mutations that were present in the later isolates were consistent with higher resistance of the bacteria to antibiotics.

**Table 2.** Non-synonymous changes in genes associated with adaptation to the lung environment, relative to earlier isolate E-S2239-16*

Gene	Description	E-MSB2494	E-MSB3405	L-001-1A	L-001-1B	L-001-1C	L-001-2A	L-001-2B	L-001-3A	L-001-3B	L-001-4	L-001-5A	L-001-5B	L-001-6
**Antibiotic resistance**														
*ftsI*	penicillin-binding protein 3			G63C		G63C R504C	G63C R504C	G63C	G63C R504C	G63C	G63C	G63C		G63C
*fusA1*	elongation factor G			G611V	G611V	G611V	G611V	G611V	G611V	G611V	G611V	G611V	G611V	G611V
*fusA2*	elongation factor G			G252V		G252V	G252V	G252V	G252V	G252V	G252V	G252V		G252V
*gyrA*	DNA gyrase subunit A	A83T		A83T	A83T	A83T	A83T	A83T	A83T	A83T	A83T	A83T	A83T	A83T
*gyrB*	DNA gyrase subunit B			E468D	E468D G89V	E468D	E468D	E468D	E468D	E468D	E468D	E468D	E468D G89V	E468D
*mexB*	RND multidrug efflux transporter MexB			643L	643L	643L	643L	643L	643L	643L	643L	643L	643L	643L
*mexZ*	transcriptional regulator MexZ			Δ1bp	Δ1bp	Δ1 bp	Δ1 bp	Δ1 bp	Δ1 bp	Δ1 bp	Δ1 bp	Δ1 bp	Δ1 bp	Δ1 bp
*mpl*	UDP-*N*-acetylmuramate: l-alanyl-gamma-d-glutamyl-meso-diaminopimelate ligase			E126	A20D	E126	E126	E126	E126	E126	E126	E126	A20D	E126
*oprD*	outer membrane porin OprD			E140	E182	E140 E182 Δ13 bp	E140 E182 Δ13 bp	E140	E140 E182 Δ13 bp	E140	E140	E140	E182	E140
**Physiology**														
*algG*	alginate-c5-mannuronan-epimerase AlgG			F398L A499T	F398L A499T	F398L A499T	F398L A499T	F398L A499T	F398L A499T	F398L A499T	F398L A499T	F398L A499T	F398L A499T	F398L A499T
*algU*	sigma factor AlgU	Q48	Δ1bp		Q30K				A193S				Q30K	
*ccoN1*	Cytochrome c oxidase cbb3-type CcoN subunit			S78	S78	S78	S78	S78	S78	D48G	S78	S78	S78	D48G
*fleQ*	transcriptional regulator FleQ			G382W	G382W	G382W	G382W	G382W	G382W	G382W	G382W	G382W	G382W	G382W
*fliA*	sigma factor FliA					D49A	D49A		D49A					
*lasR*	transcriptional regulator LasR	Q45		W195L	G31	W195L	W195L	W195L	W195L	W195L	W195L	W195L	G31	W195L
*mucA*	anti-sigma factor MucA			118Q	118Q	118Q	118Q	118Q	118Q R168C	118Q	118Q	118Q	118Q	118Q
*mucB*	negative regulator for alginate biosynthesis MucB			ins 1 bp	ins 1 bp	ins 1 bp	ins 1 bp	ins 1 bp	ins 1 bp	ins 1 bp	ins 1 bp	ins 1 bp	ins 1 bp	ins 1 bp
*mvaT*	transcriptional regulator MvaT			W107R	W107R	W107R	W107R	W107R	W107R	W107R	W107R	W107R	W107R	W107R
*pvdA*	l-ornithine N5-oxygenase	Q327								E195				
*pvdD*	pyoverdine synthetase D		C1559Y		A306S	Δ12 bp			C1559Y				A306S	
*pvdH*	l-2,4-diaminobutyrate:2-ketoglutarate 4-aminotransferase										ins 4 bp			
*pvdN*	aminotransferase PvdN								V274G	Δ1 bp				Δ1 bp
*pvdS*	sigma factor PvdS				E85								E85	
*wbpL*	glycosyltransferase WbpL		ins 1 bp	ins 1 bp	ins 1 bp	ins 1 bp	ins 1 bp	ins 1 bp	ins 1 bp	ins 1 bp	ins 1 bp	ins 1 bp	ins 1 bp	ins 1 bp

*Variants in key genes are listed here. A complete listing of gene variants is given in Table S5.

Mutations in the later isolates (Table S5; Table 2) revealed other genes with a role in adaptation of *P. aeruginosa* to the lung environment. Isolates of *P. aeruginosa* from chronic infections can lack O-antigen polysaccharide [81] and all later isolates have a frameshift mutation in *wbpL*, probably preventing O-antigen synthesis. All later isolates had mutations in the global regulators *lasR* and *mvaT* that influence a range of phenotypes, including biofilm formation and production of virulence factors. The later isolates also had mutations in the *muc* and *alg* genes that are associated with alginate production, in *fleQ* and *fliA* associated with flagella production, and in genes associated with iron acquisition such as *pvdD* and other *pvd* genes that are involved in the production of a siderophore, pyoverdine.

Although many mutations were in genes with known functions, over half of the mutations were in genes of unknown function, or for which a possible function has been assigned on the basis of sequence similarity but not experimentally investigated. It is likely that mutations in some of these uncharacterized genes contribute to the phenotypes described above, and others may contribute to different phenotypic changes associated with adaptation to the lung environment or to different micro-niches within the lung.

### Differential gene expression between an earlier and a later isolate

RNAseq of an earlier and a later isolate was carried out on bacteria grown in a synthetic CF sputum medium [43] to identify pathways and genes that had altered expression following 20 years of infection. Of 5630 genes expressed in both isolates, 34 had significantly lower expression in the later isolate and 170 had significantly higher expression (Table S7; Fig. S2). Genes of known function showing large differences in expression are summarized in Table 3. Most of the genes that have reduced expression are involved in flagellar synthesis, consistent with the presence of a mutation in the transcriptional regulator *fleQ*. The gene with the most highly reduced expression that is present in both isolates, *sfnG*, encodes an enzyme involved in oxygenation of dimethylsulfone [82], but the biological significance of its reduced expression in the context of infection in CF is not clear.

**Table 3.** Differentially expressed genes between earlier isolate E-S2239-16 and later isolate L-001-1C*

Gene	Log_2_ fold-change	FDR adjusted *P*-value	Gene description
***Reduced expression in later isolate***			
**Motility**			
*flgG*	−3.79	1.44×10^−03^	flagellar basal-body rod protein FlgG
*flgF*	−3.71	3.82×10^−03^	flagellar basal-body rod protein FlgF
*flgH*	−3.43	8.79×10^−04^	flagellar L-ring protein precursor FlgH
*flgI*	−3.34	2.46×10^−05^	flagellar P-ring protein precursor FlgI
*flgK*	−3.16	3.82×10^−04^	flagellar hook-associated protein 1 FlgK
*fleR*	−2.97	1.54×10^−04^	two-component response regulator
*flgJ*	−2.91	9.16×10^−05^	flagellar protein FlgJ
*fliF*	−2.68	4.18×10^−07^	flagella M-ring outer membrane protein precursor
**Physiology**			
*sfnG*	−4.93	3.01×10^−20^	FMNH2-dependent monooxygenase, SfnG
*mexI*	−2.85	1.05×10^−04^	RND efflux transporter
***Increased expression in later isolate***			
**Nutrient acquisition and utilization**			
*aceA*	4.14	9.76×10^−08^	isocitrate lyase AceA
*fptA*	2.95	3.85×10^−03^	Fe(III)-pyochelin outer membrane receptor
*pchA*	2.61	5.51×10^−03^	salicylate biosynthesis isochorismate synthase
*pchB*	2.45	3.67×10^−06^	salicylate biosynthesis protein PchB
*pchC*	2.77	1.98×10^−06^	pyochelin biosynthetic protein PchC
*pchD*	3.01	9.99×10^−04^	pyochelin biosynthesis protein PchD
*pchG*	2.27	2.10×10^−03^	pyochelin biosynthetic protein PchG
*rpmE2*	4.69	9.94×10^−08^	Zinc-independent paralog of ribosomal L31 protein
*dksA2*	4.23	3.17×10^−12^	transcriptional regulator
*zrmD*	3.10	1.13×10^−17^	secretion of zinc metallophore
*zrmC*	2.93	1.50×10^−03^	biosynthesis of zinc metallophore
*zrmB*	4.26	1.59×10^−11^	biosynthesis of zinc metallophore
*zrmA*	4.40	4.56×10^−11^	outer membrane receptor for zinc metallophore
**Cell surface and adhesion**			
*algA*	3.34	7.36×10^−07^	phosphomannose isomerase / guanosine 5'-diphospho-d-mannose pyrophosphorylase
*algD*	2.43	1.705×10^−03^	GDP-mannose 6-dehydrogenase AlgD
*cdrA*	2.28	2.53×10^−03^	cyclic diguanylate-regulated TPS partner A
*lecB*	3.02	2.86×10^−05^	fucose-binding lectin PA-IIL
*lptF*	3.80	1.21×10^−06^	lipotoxin F, LptF
*mucB*	2.80	3.16×10^−07^	negative regulator for alginate biosynthesis MucB
**Pathogenicity**			
*aprA*	3.24	2.44×10^−04^	alkaline metalloproteinase
*hcp1*	2.67	9.39×10^−15^	Hcp1
*tagQ1*	2.71	1.02×10^−09^	TagQ1
*tssB1*	2.25	1.71×10^−06^	TssB1
*tssC1*	2.44	1.56×10^−07^	TssC1
**Antibiotic resistance**			
*ampC*	2.97	2.42×10^−09^	β-lactamase
*mexZ*	2.73	5.11×10^−09^	negative regulator of *mexXY* efflux pump genes MexZ
**Stress response**			
*ibpA*	3.50	3.63×10^−13^	heat-shock protein IbpA
*katE*	2.98	1.44×10^−07^	catalase HPII
*lexA*	3.62	2.48×10^−06^	repressor protein LexA
*sulA*	3.38	4.52×10^−04^	SulA
**Lifestyle regulation**			
*pqsA*	2.48	1.02×10^−05^	PqsA
*pqsB*	2.32	1.34×10^−03^	PqsB
*pqsC*	2.18	3.40×10^−04^	PqsC
*pqsD*	2.44	7.65×10^−06^	3-oxoacyl-[acyl-carrier-protein] synthase III
*pqsE*	2.29	8.34×10^−05^	quinolone signal response protein
*sbrI*	1.51	2.51×10^−03^	SbrI
*sbrR*	1.33	1.43×10^−03^	SbrR

*RNAseq was carried out on earlier isolate E-S2239-16 and later isolate L-001-1C. A complete listing of all genes is given in Table S7.

Genes that had significantly increased expression in the later isolate reveal a wide range of functions associated with host–pathogen interactions, including genes involved in nutrient acquisition. Amongst those with the highest increase in expression are multiple genes involved in zinc scavenging (*zrmA-D,* also known as *cntI-O*) and the zinc starvation response (*dksA2* and *rpmE2*), consistent with high levels of expression of these genes by *P. aeruginosa* during chronic lung infection in CF [83]. The *pch* and *fptA* genes required for synthesis and uptake of an iron-scavenging siderophore, pyochelin, also had higher expression in the later isolate. The *aceA* gene that encodes isocitrate lyase also had higher expression in the later isolate, consistent with the key role of this gene in utilization of acetate and fatty acids as carbon sources [84].

As well as genes involved in nutrient acquisition and utilization, multiple genes involved in biofilm formation and adhesion to host cells had higher expression in the later isolate. These include genes associated with synthesis of cell-surface molecules, including *lecB* (lectin), *cdrA* (an adhesin) and *lptF* (an outer membrane protein associated with host cell attachment). Some though not all genes involved in alginate production also had higher expression in the later isolate.

Several genes associated with stress responses (*lexA*, *sulA*, *katE, pfpI* and *ibpA*) had higher expression in the later isolate, probably reflecting at least in part ongoing exposure of the bacteria to the host immune system. Perhaps as part of this response, or perhaps reflecting interactions with other bacteria, some Type VI secretion system genes (*tssB1*, *tssC1*, *hcp1* and *tagQ1*) as well as the protease-encoding *aprA* gene had higher expression in the later strain. The complex nature of bacterial adaptation to the environment of the host lung is also shown by the higher expression of genes required for synthesis of the *Pseudomonas* quinolone signal (PQS). PQS is a quorum sensing molecule that contributes to regulation of a range of different functions, but is also cytotoxic, alters the host immune response and contributes directly to iron acquisition [85]. The SbrIR sigma/antisigma factor system that controls swarming activity also had higher expression in the later isolate.

Altered gene expression can also contribute to resistance of *P. aeruginosa* to antibiotics. Isolates of *P. aeruginosa* from chronically infected patients often have increased expression of the *ampC* gene, which is associated with resistance to cephalosporins such as ceftazidime. This is true of the later isolate, which had increased expression of *ampC*. A major factor in the resistance of *P. aeruginosa* to antibiotics is increased expression of efflux pump genes [6]. None of the efflux genes had significantly higher expression in the later isolate. The *mexXY* genes had increased expression, consistent with the presence of a mutation in the regulator gene *mexZ*, but this did not reach statistical significance (Table S7).

Transcriptomic differences between the earlier and later isolate demonstrate the wide range of changes that *P. aeruginosa* undergoes while adapting to the host lung environment. It is important to note that the majority of the 170 genes that had significantly higher expression in the later isolate, including some of those with the largest differences in expression, do not have well-defined functions. Intriguingly, two of the genes showing highest increase in expression are a non-coding RNA of unknown function (P8) and a tRNA gene (PA4581.1), but the biological significance of this is not known.

### Phenotypic changes between earlier and later isolates

The patient was treated with a wide range of antibiotics during infection (Tables S1 and S2). Genome analysis (Table 2; Table S5) showed that later isolates had acquired mutations likely to increase the ability of the bacteria to tolerate carbapenem, fluoroquinolone, cephalosporin and aminoglycoside antibiotics. Resistance to meropenem (a carbapenem), ciprofloxacin (a fluoroquinolone), ceftazidime (a cephalosporin) and tobramycin (an aminoglycoside) was therefore tested for all isolates. Of the 11 later isolates, four were clinically resistant to all antibiotics tested (Table 1). Only two later isolates were not resistant to more than one class tested. Earlier isolates were all sensitive to tobramycin and meropenem and were inhibited by lower concentrations of ciprofloxacin than later isolates.

**Fig. 2.**
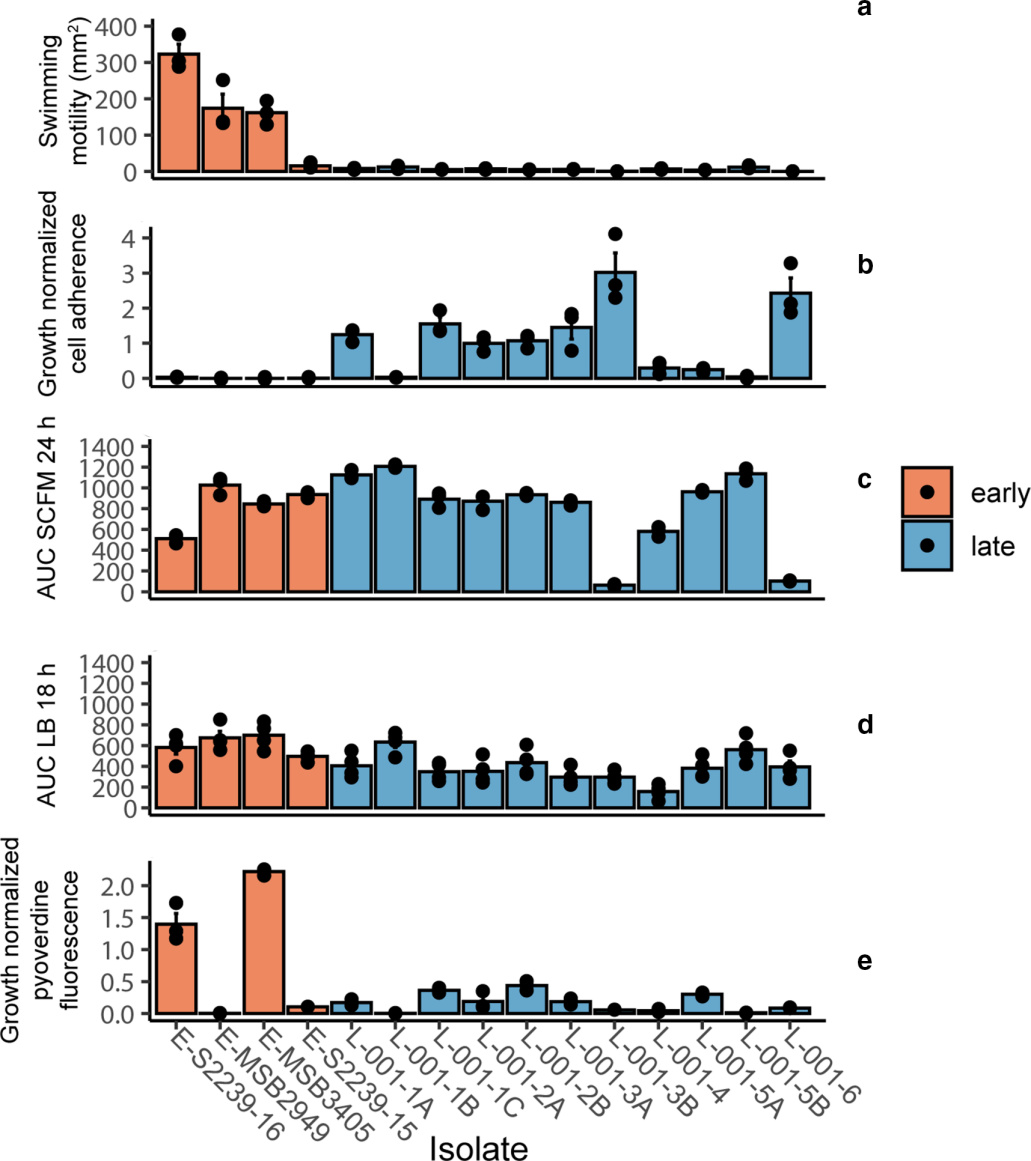
Phenotypic analysis of isolates. Earlier samples are shown in orange, and later in blue. Three biological replicates were carried out for each experiment, with each point representing a biological replicate and median values shown as crossbars. A one-way ANOVA with post-hoc Dunnett’s test was carried out between early and late isolates, and Bonferroni-corrected *P*-values are stated. (**a**) Swimming motility of isolates on M8 media containing 0.3 % agar. ***P=4.12×10^−13^. (**b**) Cell adherence in a microtitre plate assay. Crystal violet staining of standing cultures was normalized to culture OD_600_. *P=0.034. (**c**) Growth of isolates in synthetic cystic fibrosis media (SCFM), summarized as area under the curve (AUC). *P=0.045. (**d**) Growth of isolates in LB broth. ns, No significant change. (**e**) Pyoverdine production normalized to culture OD_600_ following growth in King’s B broth. ***P=8.96×10^−7^.

*P. aeruginosa* commonly adapts to the host lung environment through altered biofilm formation, reflected in altered adherence properties and associated with reduced motility [4, 26]. None of the later isolates showed swimming motility whereas three of the four ancestral isolates were motile (Fig. 2a). Nine of the 11 later isolates also showed increased adherence in an *in vitro* assay, whereas all earlier isolates showed no detectable adherence (Fig. 2b).

Following long-term infection, *P. aeruginosa* can have altered growth in laboratory culture due to development of auxotrophy or of small colony variants [86, 87]. Isolates were tested for growth in nutrient-defined SCFM medium that is representative of the chemical composition of CF sputum [43]. Three of the later isolates showed reduced growth in SCFM compared to earlier isolates (Fig. 2c). There was no clear pattern of difference in growth between the earlier and later isolates in nutrient-rich medium, although one later isolate grew more slowly than any other (Fig. 2d).

During the early stages of infection in CF patients *P. aeruginosa* acquires iron by secreting a siderophore, pyoverdine, but during chronic infections the bacterium becomes less dependent on pyoverdine, transitioning to utilizing haem and other iron sources [88–91]. Earlier and later isolates were tested for pyoverdine production in an iron-limited growth environment. Two earlier isolates showed high levels of pyoverdine production. All later isolates, and one earlier isolate, showed impaired production of pyoverdine, consistent with reduced dependence on pyoverdine-mediated iron acquisition as infection progresses (Fig. 2e).

## Discussion

The availability of isolates of *P. aeruginosa* from the same patient over a 20 year timeframe has allowed us to integrate genomic, transcriptomic and phenotypic approaches to shed light on the changes that occur in these bacteria during prolonged infection. All of the later isolates in our study shared a high amount of genetic similarity with three of the earlier isolates, making it extremely likely that the later isolates are descended from a common ancestor closely related to the earlier group and indicating that a single lineage was able to maintain an infection over more than 20 years (Fig. 1a; Fig. S1). However, a genetically distinct co-infecting earlier isolate was not represented in the later isolates and was probably lost from the infection. These findings are consistent with studies from geographically separate centres [19–21], showing that CF patients acquire a colonizing strain of *P. aeruginosa* that evolves throughout the course of infection. Many of the pathways that underwent mutational change in the patient in this study have also undergone evolutionary change during chronic *P. aeruginosa* infections in other CF patients [20, 21, 26, 30].

Genome sequencing data indicate that a population bottleneck occurred during infection. All later isolates contain an identical 125 kb deletion not present in earlier isolates (Fig. 1b) and many genes in these isolates had identical alleles that are different from any of the ancestral isolates (Table 2, Table S5). This bottleneck could have occurred due to a major but temporary reduction in the number of infecting bacteria, or because a mutation arose that conferred a significant competitive advantage. Genetic bottlenecks, which significantly reduce genetic diversity within a population, have not been reported in other longitudinal studies of *P. aeruginosa* genome evolution during chronic infection [20–22, 30] and it remains to be determined how often they occur. Nonetheless the later isolates have a significant degree of genetic diversity (Table S5, Table S6), consistent with diversification of *P. aeruginosa* after the bottleneck event and with generation of different morphotypes and adaptation to micro-niches during chronic infection within the CF lung [23, 29, 30, 92]. In comparison, the genetically related earlier isolates have relatively little genetic diversity, consistent with a shorter amount of time for adaptation to the lung environment. Deletions have recently been identified as occurring at a significant frequency in clinical isolates of *P. aeruginosa *, with up to 15 % of isolates containing large deletions [9, 32, 33, 93]. Large deletions in the *P. aeruginosa* genome can contribute to phage resistance [94] and to resistance to carbapenems and other β-lactam-based antibiotics [14, 95]. The 125 kb deletion identified here overlaps with the deleted regions identified in earlier studies but whether this deletion confers resistance to phage or antibiotics has not yet been determined. The 0.9 Mb inversion of part of the genome in some of the later isolates also represents a major genome rearrangement. The occurrence of genome inversions during chronic infection has been little studied, although there is one previous report [69]. How often such inversions occur, and how they affect the phenotype of infecting bacteria, remains to be determined.

The mean mutation rate for *P. aeruginosa* during infection in CF has been estimated to be between 2.6 and 10 mutations per year [19, 21] but this rate is higher in strains with defects in DNA repair (so-called ‘hypermutators’) [96]. Later isolates contain a different variant than earlier isolates of the MutY protein that normally reduces the incidence of GC→TA transversions [70]. The relatively high mutation rate of *P. aeruginosa* in this patient is probably due to reduced activity of MutY, with the consequent increase in mutation rate facilitating an increased rate of genetic diversification and adaptation of the bacteria during the course of the infection. One of the genes with the highest increases in expression in the later isolate is the anti-mutator gene *pfpI* that is upregulated in response to *mutY* mutations [97], consistent with the MutY variant present in later isolates being disfunctional.

Transcriptomic profiling of an earlier and a later isolate showed that substantial changes in gene expression occurred during the course of infection. This contrasts with the high similarity in transcriptomic profiles of different isolates of *P. aeruginosa* collected at the same time from a CF lung [98]. Many of the genes with higher expression in the later isolate have been noted in studies comparing *P. aeruginosa* in laboratory culture with those in late-stage infections within CF lungs [99, 100]. Although many of the genes with altered expression levels in the later isolate have known functions, a large proportion of these genes have undefined or unconfirmed functions consistent with other studies and demonstrating the complex nature of CF lung-adapted *P. aeruginosa* [98–100]. Most of the other genes with significantly reduced expression have no known function, emphasizing our incomplete understanding of how *P. aeruginosa* adapts to the lung environment.

During infection *P. aeruginosa* became resistant to all the antibiotic classes routinely used in treatment of the patient (Table 1) and both genome and transcriptome analysis were used to understand the basis of resistance. Resistance arose through mutations, with no evidence of acquisition of antibiotic resistance genes. All later isolates have non-synonymous mutations in resistance-related genes including *gyrB*, *fusA1*, *mpl* and *oprD* that are associated with resistance to fluoroquinolones, aminoglycosides, cephalosporins and carbapenems, respectively. The later isolate also had significantly increased expression of *ampC*, a change that is associated with increased resistance to cephalosporins. It is likely that increased expression is due to mutations in *mpl* that are present in all later isolates, as mutations in this gene increase expression of *ampC* and contribute to ceftazidime resistance [14, 101]. Antibiotic resistance in *P. aeruginosa* is also commonly associated with increased expression of efflux pump genes [6], but we did not observe widespread differences in efflux gene expression between an earlier and a later isolate. One exception was expression of the *mexXY* efflux-encoding operon that had increased expression, albeit not significant, in the later isolate consistent with the presence of a frameshift mutation in the *mexZ* repressor-encoding gene. Expression of *mexXY* genes is highly varied in *P. aeruginosa* isolated from patients with CF due in part to mutations in *mexZ*, which are associated with aminoglycoside resistance [102–104]. Curiously, early isolates contain a premature stop codon in *mexB* that encodes a component of the MexAB-OprM efflux pump, which is involved in resistance of *P. aeruginosa* to a range of antibiotics [105, 106]. This stop codon has undergone mutation to a leucine codon in later isolates, potentially reflecting selection for a functional efflux pump. Our findings demonstrate the multifactorial nature of antibiotic resistance development during chronic infection in the CF lung, with a complex interplay between mutations and changes to gene expression.

All the later isolates in our study had lost flagellar-mediated motility (Fig. 2a), a phenotype commonly associated with biofilm formation and avoidance of the host immune system. Transcriptomic analysis demonstrated a significant reduction of expression of flagellar genes in a later isolate (Table 3; Table S7) consistent with a lack of motility. All of the later isolates have a mutation in *fleQ* that encodes a sigma factor required for expression of flagellar synthesis genes [107], providing a molecular explanation for loss of motility. Most of the later isolates showed enhanced adherence in a microtitre plate assay, a common property of *P. aeruginosa* isolated from chronic infections [108]. The genetic basis for this complex phenotype is not clear, although it is noteworthy that a number of genes that may influence biofilm formation, including global regulators (PQS, Las and Sbr) and cell surface adhesins (LecB and CdrA), had higher expression in the later isolate. Later isolates also had mutations in genes required for synthesis of lipopolysaccharide, Pel polysaccharide and alginate, all of which might influence attachment and biofilm formation.

*P. aeruginosa* from chronic infections often produce copious amounts of extracellular alginate due to mutations in regulatory genes such as *mucA* and *mucB* that control the alginate synthesis pathway [109], but the only isolate to have a mucoid phenotype was the early isolate E-S2239-16. Indeed, a premature stop codon in *mucA* in that strain has been replaced by a glutamine-coding codon that would restore production of full-length protein with potential to repress alginate gene expression in later isolates. All later isolates also have mutations in the alginate regulatory gene *mucB* and in some cases the regulatory gene *algU*, as well as mutations in genes encoding alginate-encoding enzymes required for alginate synthesis. Expression of some alginate synthesis genes was upregulated in the later isolate although this did not result in a mucoid phenotype. The relationship between alginate genotype, gene expression and phenotype is clearly complex in the isolates studied here.

Host colonization is also influenced by quorum sensing, which controls production of a range of virulence factors. Later isolates had mutations in the quorum-sensing regulator *lasR* that is often mutated in chronically infected CF patients [4, 110–112], and expression of the LasR-regulated *apr* gene that encodes alkaline protease was higher in the later isolate. Expression of the *pqs* genes that encode the PQS quorum sensing molecule, which regulates a wide range of infection-associated phenotypes [112], was also higher in the later isolate. Later isolates also contained a mutation in *mvaT* that is associated with quorum sensing and virulence factor production, as well as with biofilm formation [113, 114]. Clearly, quorum sensing pathways evolve during chronic infection, although the complex interplay between quorum sensing pathways mean that further work will be needed to dissect out the effects of these changes on phenotype.

Adaptation to the host environment was also reflected in metabolic changes. The later isolate had much higher expression of isocitrate lyase, a key enzyme in the glyoxylate pathway, reflecting the use of lipids as a carbon source during infection [84]. During infections, *P. aeruginosa* counters zinc starvation by upregulating expression of zinc acquisition genes [83], and these genes were amongst those with the greatest increase in expression in the later isolate. *P. aeruginosa* has multiple mechanisms to acquire iron. Genes for synthesis of an iron-scavenging siderophore, pyochelin, also had higher expression in the later isolate, indicating that pyochelin has an important role in iron acquisition during the later stages of infection. Conversely, during infection in the CF lung, *P. aeruginosa* transitions away from the use of the siderophore pyoverdine for iron acquisition, instead using other sources of iron [88–91]. Two of the earlier isolates but none of the later isolates have a high production of pyoverdine (Fig. 2e). The later isolates have mutations in different pyoverdine genes (Table S5), indicating that loss of pyoverdine production has occurred independently on several occasions although transcription of *pvd* genes was similar in the earlier and later isolate (Table S7).

In conclusion, this study reveals the relationship between genomic, transcriptomic and phenotypic changes undergone by *P. aeruginosa* during long-term infection in the lungs of a person with CF. Adaptations included a trend towards increased antibiotic resistance, altered nutrient acquisition and altered biofilm production, and were accelerated through an increased mutation rate. While many of the genomic and transcriptomic alterations affect known phenotypes, many affect genes with no known function, showing that an understanding of how *P. aeruginosa* evolves during infection in CF is still far from complete. Comparable studies of infections in other patients, combined with experimental and bioinformatic approaches, can be expected to shed further light on how *P. aeruginosa* adapts and maintains infections in the CF lung.

## Supplementary Data

Supplementary material 1

Supplementary material 2

Supplementary material 3
