# Supplementary material for: Genome evolution drives transcriptomic and phenotypic adaptation in Pseudomonas aeruginosa during 20 years of infection
Source: Microb Genom. 2021 Nov 26;7(11):000681. doi: 10.1099/mgen.0.000681 (PMC8743555; doi:10.1099/mgen.0.000681)
Supplement: Supplementary material 1 [file mgen-7-0681-s001.pdf]

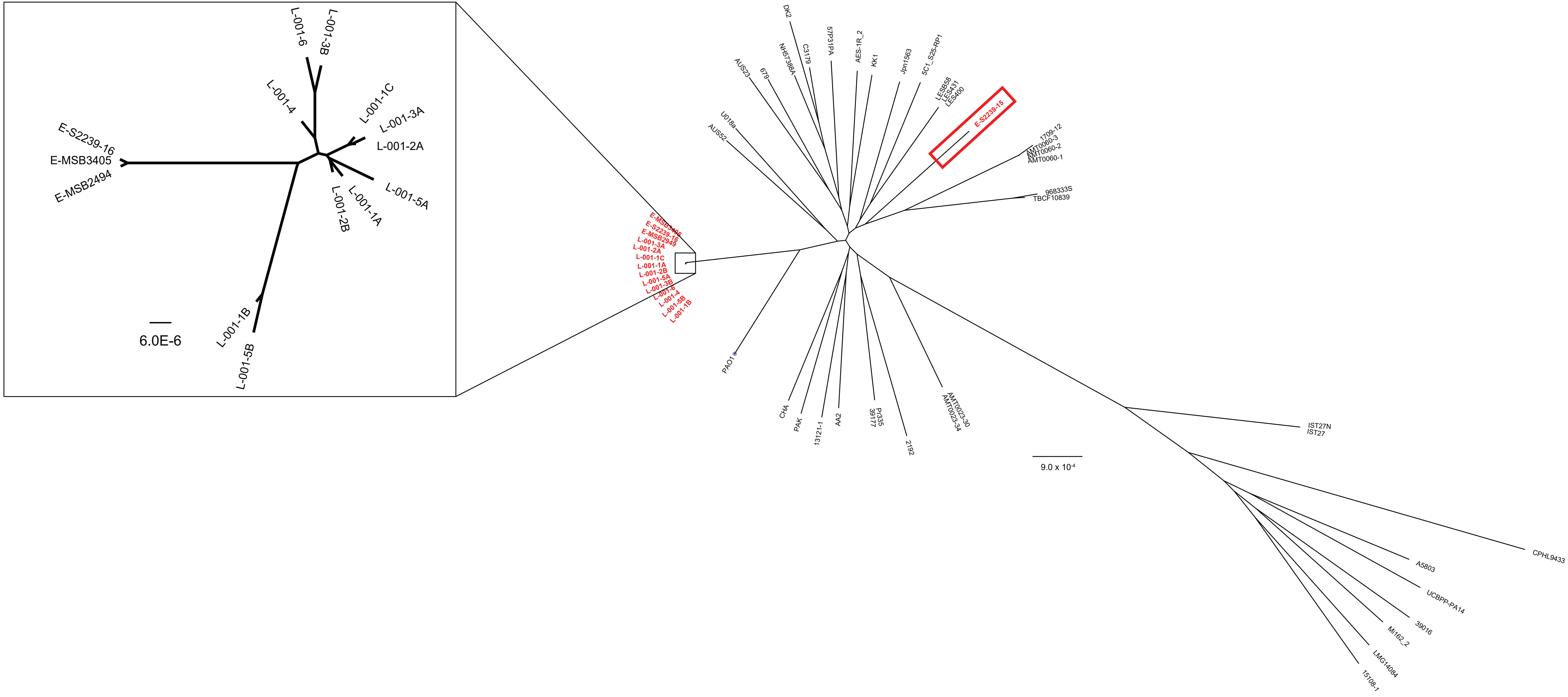

S1 Fig. Phylogenetic tree of isolates used in this study. Clinical isolates of *Pseudomonas aeruginosa* obtained from a person with cystic fibrosis shown in red. Reference strains of *Pseudomonas aeruginosa* are shown in black and were chosen to represent the common genetic diversity within *P. aeruginosa* species from (De Soyza et al., 2013. *MicrobiologyOpen,* **2**(6) 1010-1023). Zoomed in area shows the positioning of the closely related patient isolates excluding the distant E-S2239-15 isolate.

S1 Table. Summary of isolates taken for study, patient details and antibiotic treatment at time of sampling.

Sample	Date of Collection (MM/DD/YY)	Patient age	FEV1 % predicted [a] at sputum sampling	Antibiotic treatment at time of sampling
		**Early Isolates**		
E-S2239-16<br>E-MSB2494<br>E-MSB3405<br>E-S2239-15	12/91	26	53 [b]	Cotrimoxizole, Ciprofloxacin, Gentamycin
		**Late Isolates**		
L-001-1A<br>L-001-1B<br>L-001-1C	15/08/12	46	18	Azithromycin
L-001-2A<br>L-001-2B	17/10/12	47	21	Azithromycin, Tobramycin, Cefepime
L-001-3A<br>L-001-3B	21/11/12	47	21	No Data
L-001-4	21/01/13	47	17	Tobramycin, Meropenem
L-001-5A<br>L-001-5B	18/04/13	47	17	No Data
L-001-6	06/05/13	47	17	Tobramycin, Ceftazidime, Azithromycin

[a] Sinclair SW, Avery SF, Brady DM, Smith DA, Holst PE, O'Donnell TV. Prediction formulae for normal pulmonary function values in New Zealand European subjects. N Z Med J. 1980;91(651):1-5.

FEV1% predicted is defined as the Forced Expiratory Volume of the patient divided by the average FEV1% within the population for any person of similar age, sex, and body composition. This is used as a measure of reduced lung function, compared to what would be expected of a person with 'normal' lung function.

[b] Precise FEV1 is not available at time of sampling, number presented is average FEV1 % for the 2nd half of 1991

S2 Table. Summary of antibiotics administered to the patient at time of, or between sampling of early and late isolates of *P. aeruginosa*.

**Known antibiotic treatments given (1991-2013) [a, b]**
Augmentin
Azithromycin
Cefepime
Ceftazidime
Ciprofloxacin
Clarithromycin
Colomycin/Colistin
Cotrimoxazole
Fucidin
Gentamycin
Meropenem
Moxifloxacin
Piperacillin
Tobramycin

[a] *This is not an exhaustive list of all antibiotics the patient received, but covers those administered at time of sampling or between samples.*

[b] *Antibiotics listed are ones the patient received, this is not a list of antibiotics administered solely to combat P. aeruginosa infection.*

S3 Table. Summary assembly and gene prediction statistics for isolates whole-genome sequenced.

isolate	total_length	n_contigs	circular	N_count	gaps	ont_N50	ont_qscore	ont_bases	ont_cov	miseq_qscore	miseq_bases	miseq_cov	pred_CDS	Biosample
E-S2239-15	6,407,559	1	yes	0	0	19,875	13.3	1,394,937,072	217.7	35.3	103,038,935	16.1	5802	SAMN13234651
E-S2239-16	6,315,896	1	yes	0	0	23,364	13.3	1,146,375,768	181.5	35.3	114,392,328	18.1	5717	SAMN13234648
E-MSB2494	6,330,120	1	yes	0	0	7,556	13.1	1,001,416,848	158.2	35.3	154,738,111	24.4	5737	SAMN13234649
E-MSB3405	6,315,897	1	yes	0	0	22,478	13.3	1,196,529,488	189.4	35.3	96,322,838	15.3	5718	SAMN13234650
L-001-1A	6,269,726	1	yes	0	0	20,311	13.3	824,514,262	131.5	35.3	71,982,494	11.5	5691	SAMN13234657
L-001-1B	6,270,234	1	yes	0	0	26,365	13.3	658,327,564	105.0	35.3	118,980,668	19.0	5705	SAMN13234652
L-001-1C	6,268,635	1	yes	0	0	17,303	13.3	1,290,121,361	205.8	35.2	96,909,163	15.5	5688	SAMN13234659
L-001-2A	6,268,813	1	yes	0	0	19,144	13.3	1,139,680,807	181.8	35.3	84,017,737	13.4	5691	SAMN13234660
L-001-2B	6,270,419	1	yes	0	0	19,382	13.2	1,173,114,665	187.1	35.3	97,922,867	15.6	5695	SAMN13234658
L-001-3A	6,267,105	1	yes	0	0	19,255	13.3	1,084,955,690	173.1	35.2	97,672,750	15.6	5692	SAMN13234661
L-001-3B	6,269,926	1	yes	0	0	17,568	13.3	719,291,575	114.7	35.3	117,042,558	18.7	5699	SAMN13234655
L-001-4	6,269,046	1	yes	0	0	20,001	13.2	697,520,061	111.3	35.3	113,721,808	18.1	5692	SAMN13234654
L-001-5A	6,270,090	1	yes	0	0	22,496	13.3	626,823,565	100.0	35.1	112,667,053	18.0	5696	SAMN13234662
L-001-5B	6,270,238	1	yes	0	0	20,014	13.2	993,479,333	158.4	35.3	100,880,789	16.1	5709	SAMN13234653
L-001-6	6,255,746	1	yes	0	0	17,642	13.3	815,860,744	130.4	35.3	113,412,261	18.1	5681	SAMN13234656

LEGEND	
**isolate**	Strain IDs
**total_length**	Total length (bp) of contigs in the assembly
**n_contigs**	Number of contigs (if 1, then one chromosome w/ plasmid)
**circular**	Is contig 1 (chromosome) complete and circular (yes/no)
**N_count**	Number of uncalled nucleotides (N's) in final sequence)
**gaps**	Amount of gaps (stretches of N's) in the assembly
**ont_N50**	Read length that delimits 50% of sequenced bases (Nanopore)
**ont_qscore**	Mean Phred quality score of Nanopore (long) reads
**ont_bases**	Total amount of bases sequenced per nanopore library
**ont_cov**	Amount of nanopore bases divided by genome size
**miseq_qscore**	Mean Phred quality score of Illumina MiSeq (short) reads
**miseq_bases**	Total amount of bases sequenced per MiSeq library
**miseq_cov**	Amount of MiSeq bases divided by genome size
**pred_CDS**	Number of predicted protein coding genes (from Prokka)

S4 Table. Summary mapping statistics for RNAseq of early isolate E-S2239-16 and late isolate L-001-1C

Sample	Number of paired reads	Sequence length range	Number of bootstraps	Mapping (%)
E-S2239-16				
01	3446702	20-125	1000	80.4
02	4903036	20-125	1000	86.6
03	4142012	20-125	1000	83.9
L-001-1C				
01	5021309	20-125	1000	80.6
02	4928204	20-125	1000	85.7
03	4994328	20-125	1000	83.5

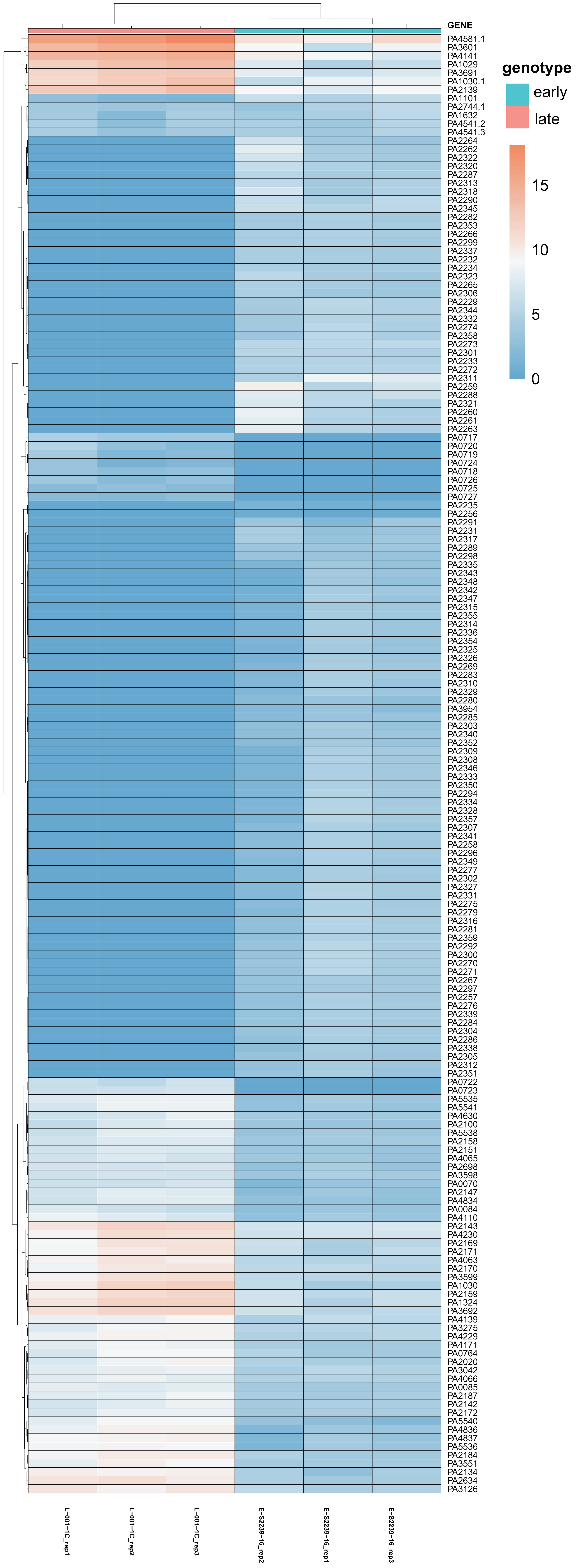

S2 Fig. Heatmap showing significantly differentially expressed genes from RNAseq of L-001-1C and E-S2239-16. Expression quantified as log of the counts per gene. Triplicates of L-001-1C RNAseq are shown in the left columns, triplicates of E-S2239-16 RNAseq are shown in the right columns.

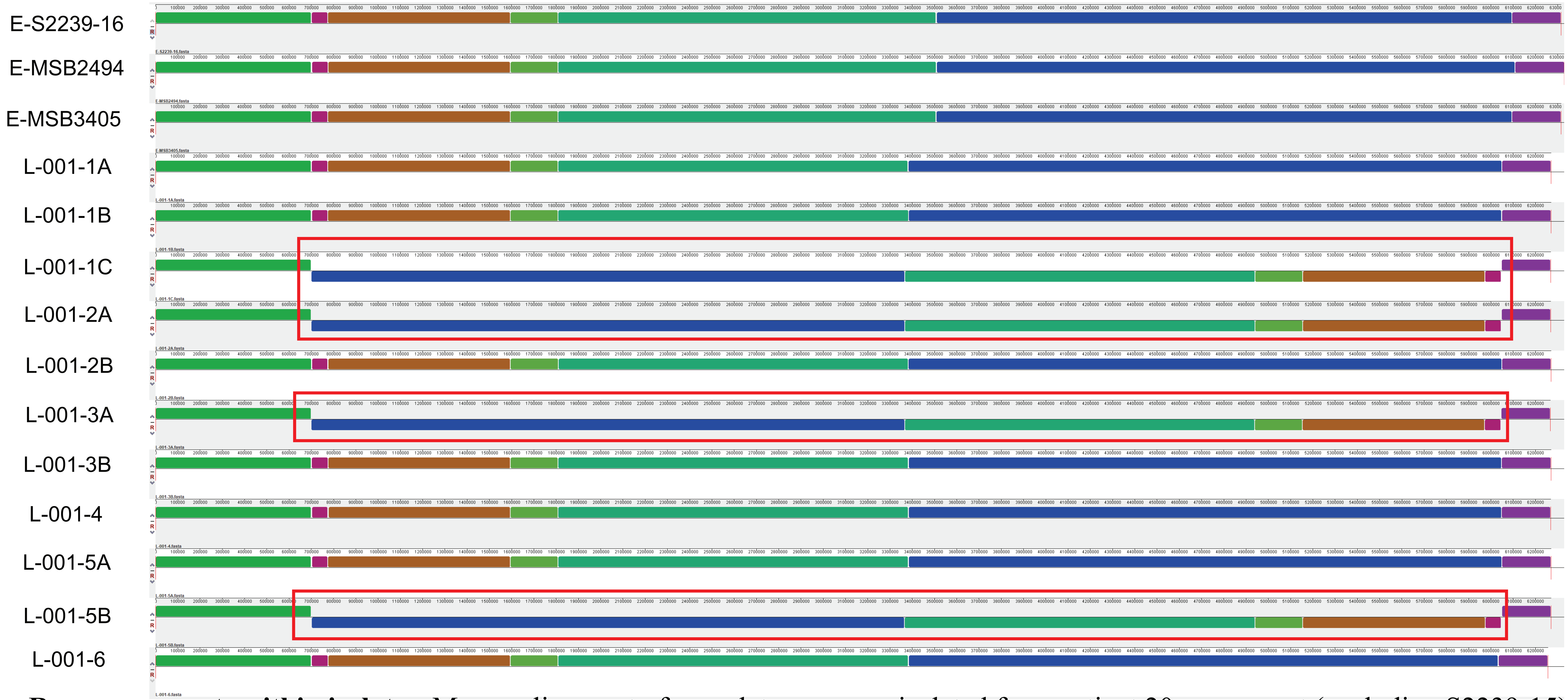

S3 Fig. Rearrangements within isolates. Mauve alignment of complete genomes isolated from patient 20 years apart (excluding S2239-15). Showing a clear inversion of 0.9Mb (highlighted) in 4 of the 11 late isolates, occuring between rRNA operons which are the inversion endpoints.

S6 Table. Pairwise matrix of non-synonymous changes between isolates of *P. aeruginosa* from patient with CF

	E-S2239-16	MSB2494	MSB3464	L-001-1A	L-001-1B	L-001-1C	L-001-2A	L-001-2B	L-001-3A	L-001-3B	L-001-4	L-001-5A	L-001-5B	L-001-6
**E-S2239-16**	0	12	4	179	246	211	209	197	214	245	206	216	266	239
**E-MSB2494**	-	0	12	185	252	219	215	205	220	251	212	222	272	245
**E-MSB3464**	-	-	0	179	246	213	209	199	214	245	206	216	266	239
**L-001-1A**	-	-	-	0	167	46	44	30	49	94	51	49	185	86
**L-001-1B**	-	-	-	-	0	195	191	183	198	229	190	198	40	221
**L-001-1C**	-	-	-	-	-	0	16	64	23	124	81	77	213	114
**L-001-2A**	-	-	-	-	-	-	0	62	13	122	79	75	211	112
**L-001-2B**	-	-	-	-	-	-	-	0	69	112	71	65	203	106
**L-001-3A**	-	-	-	-	-	-	-	-	0	127	86	82	218	119
**L-001-3B**	-	-	-	-	-	-	-	-	-	0	81	127	247	52
**L-001-4**	-	-	-	-	-	-	-	-	-	-	0	84	208	71
**L-001-5A**	-	-	-	-	-	-	-	-	-	-	-	0	216	117
**L-001-5B**	-	-	-	-	-	-	-	-	-	-	-	-	0	239
**L-001-6**	-	-	-	-	-	-	-	-	-	-	-	-	-	0

Number of non-synonymous changes

0
10
50
100
200
300

S8 Table. Prevalence of specific nucleotide changes across all single nucleotide in all *P. aeruginosa* isolates analysed in this study

Mutant allele (freq)

Starting allele	Single nucleotide mutation (n=935)	A	T	G	C
	A	0	2	22	1
	T	4	0	6	19
	G	49	298	0	4
	C	326	54	3	0

Mutant allele (%)

Starting allele	Single nucleotide mutation (n=935)	A	T	G	C
	A	0.0	0.3	2.8	0.1
	T	0.5	0.0	0.8	2.4
	G	6.2	37.8	0.0	0.5
	C	41.4	6.9	0.4	0.0

freq	(%)
0	0
10	1
50	5
100	10
200	20
400	40
